# Comprehensive analysis and discovery of drought-related NAC transcription factors in common bean

**DOI:** 10.1186/s12870-016-0882-5

**Published:** 2016-09-07

**Authors:** Jing Wu, Lanfen Wang, Shumin Wang

**Affiliations:** Key Laboratory of Crop Germplasm Resources and Utilization, Ministry of Agriculture, The National Key Facility for Crop Gene Resources and Genetic Improvement, Institute of Crop Science, the Chinese Academy of Agricultural Sciences, Beijing, 100081 China

**Keywords:** Common bean, Transcription factors, Drought

## Abstract

**Background:**

Common bean (*Phaseolus vulgaris* L.) is an important warm-season food legume. Drought is the most important environmental stress factor affecting large areas of common bean via plant death or reduced global production. The NAM, ATAF1/2 and CUC2 (NAC) domain protein family are classic transcription factors (TFs) involved in a variety of abiotic stresses, particularly drought stress. However, the NAC TFs in common bean have not been characterized.

**Results:**

In the present study, 86 putative NAC TF proteins were identified from the common bean genome database and located on 11 common bean chromosomes. The proteins were phylogenetically clustered into 8 distinct subfamilies. The gene structure and motif composition of common bean NACs were similar in each subfamily. These results suggest that NACs in the same subfamily may possess conserved functions. The expression patterns of common bean NAC genes were also characterized. The majority of NACs exhibited specific temporal and spatial expression patterns. We identified 22 drought-related NAC TFs based on transcriptome data for drought-tolerant and drought-sensitive genotypes. Quantitative real-time PCR (qRT-PCR) was performed to confirm the expression patterns of the 20 drought-related NAC genes.

**Conclusions:**

Based on the common bean genome sequence, we analyzed the structural characteristics, genome distribution, and expression profiles of NAC gene family members and analyzed drought-responsive NAC genes. Our results provide useful information for the functional characterization of common bean NAC genes and rich resources and opportunities for understanding common bean drought stress tolerance mechanisms.

**Electronic supplementary material:**

The online version of this article (doi:10.1186/s12870-016-0882-5) contains supplementary material, which is available to authorized users.

## Background

Common bean (*Phaseolus vulgaris* L.) is one of the most important crops worldwide and plays important roles in resolving food shortages in Africa and adjusting diet structure in developed countries. However, the growth and productivity of common bean are severely affected by abiotic stress, particularly drought stress. Drought affects large areas of common bean in China by causing plant death or reducing production. Preventing loss over the next few decades is already a challenge in China, particularly in the provinces of Xinjiang and Shanxi. Thus, it is very important to identify drought-associated genes in the common bean germplasm.

Transcription factors (TFs) are pivotal regulators involved in the response to abiotic stresses such as drought, salt, and cold [1–5]. A total of 129,288 TFs belonging to 58 different families from 83 species have been identified in the plant TF database (PlantTFDB, version 3.0) [6]. The TF family includes AP2 (1,776), ARF (1,914), and C3H (4,019), among others. The largest TF family is the bHLH family, which comprises 11,428 TFs, followed by MYB (8,746) and ERF (8,688). The species in this database represent Chlorophyta, Bryophyta, Lycopodiophyta, Coniferopsida, basal Magnoliophyta, Monocot and Eudicot. The genome of the monocot maize has the largest number of TFs, 3,316 (2,231 loci), which are classified into 55 families. Approximately 10.9 % of the genome of the eudicot *Glycine max* encodes more than 5,069 TFs (3,714 loci) classified into 57 families [7].

The NAM, ATAF1/2 and CUC2 (NAC) genes are plant-specific TFs that constitute one of the largest families of plant transcription factors. NAC family genes are characterized by a conserved NAC domain at the N-terminus consisting of nearly 160 amino acid residues. The NAC domain is divided into five subdomains (A-E), and the C-terminal regions of NAC proteins are not conserved [8–15]. PlantTFDB (V3.0) contains 8,133 NAC genes from 74 species. The plant species with the most NAC genes are *Populus trichocarpa* (289), *Gossypium raimondii* (266), *Malus domestica* (253), *Glycine max* (247), and *Eucalyptus grandis* (202). By contrast, 15 plant species, including *Vigna unguiculata* (20), *Brassica oleracea* (39), and *Helianthus annuus* (21), have fewer than 50 reported NAC loci in PlantTFDB. Interestingly, there are few TFs from food legumes in PlantTFDB. Furthermore, NAC proteins have recently been reported in algae, where they may play a role in the stress response [16]. In recent years, the whole genome sequences of several food legumes have been completed, including those of pigeonpea [17], chickpea [18, 19], common bean [20, 21], mung bean [22], and adzuki bean [23]. These genome sequences provide a wonderful opportunity for a comparative genome survey of new TFs from food legumes. In plants, NAC genes regulate a variety of plant developmental processes, including floral morphogenesis [24], root development [25], leaf senescence [26, 27], stress-inducible flowering induction [28], seed development [29] and fiber development [30]. NAC domain proteins have also been implicated in plant abiotic stresses and defense responses, such as salt [31, 32], wounding [33], cold [34], and particularly drought [31, 32, 35]. For example, ANAC019, ANAC055, ANAC072 and ATAF1 regulate the expression of stress-responsive genes under drought stress in Arabidopsis [36, 37]. The wheat TaNAC29, TaNAC47, TaNAC67 and TaNAC2 genes respond to drought stress [1, 38–40]. Similarly, transgenic rice overexpressing OsNAC045, OsNAC6, and OsNAC10 exhibits enhanced resistance to drought stress [41–43]. Recently, the roles of a stress-related NAC transcription factor (MlNAC9) were reported in *Miscanthus lutarioriparius* and in improved drought-tolerant transgenic cultivars [32]. Although a large number of NAC TFs have been functionally characterized in *Arabidopsis*, wheat, rice, and other plants, the functions of the majority of NAC members remain unknown in legumes. For common bean, a model legume species, there are very limited reports on the functional characterization of NAC TFs. Recently, chickpea CarNAC3 and CarNAC5 were reported as transcriptional activators involved in the drought stress response [44, 45]. Tran et al. analyzed 31 full-length NAC genes from soybean and determined that nine were induced by drought [46]. GmNAC043, GmNAC085 and GmNAC101 were identified in drought-tolerant soybean cultivars by genetic engineering [47]. However, there have been no reports about drought-tolerant related NAC TFs from common bean.

In our study, we performed genome-wide identification of NAC domain TFs in common bean and detailed analyses of the genome distribution, gene structure, conserved motifs and expression patterns under drought stress. Our results provide a subset of potential candidate drought-tolerant related NAC genes for future analyses of gene function in common bean.

## Results

### Identification of NAC transcription factors in common bean

In this study, the Hidden Markov Model (HMM) profile of the Pfam NAC domain (PF02365) was used as a query to identify NAC genes in the common bean genome (release 1.0, https://phytozome.jgi.doe.gov/pz/portal.html#!info?alias=Org_Pvulgaris). A total of 106 non-redundant putative NAC genes were obtained, of which 86 full-length protein sequences were used for further analyses, such as gene structure and phylogenetic tree analyses. First, we analyzed the genome, CDS and protein lengths; MW; pI; and subcellular localization of these NAC genes (Additional file 1: Table S1). The genome length (from the start to stop codons) of these NAC genes ranged from 741 bp (Phvul.007G140300) to 5,751 bp (Phvul.001G161700). The CDS length ranged from 537 bp (Phvul.007G140300) to 2,016 bp (Phvul.006G087000), protein length from 179 AA (Phvul.007G140300) to 672 AA (Phvul.006G087000), MW from 20.20 kDa (Phvul.007G140300) to 76.38 kDa (Phvul.006G087000) and pI from 4.59 (Phvul.007G140500) to 9.81 (Phvul.007G140300). Subcellular localization prediction indicated that 74 genes were located in the nucleus and 12 genes were potentially extracellular.

### Genome distribution of common bean genes

**Fig. 1:**
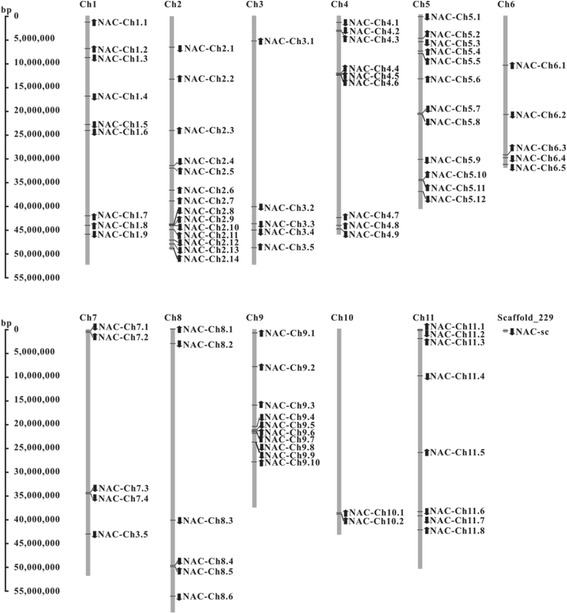
Chromosomal location of common bean NAC genes. A total of 85 NAC genes were mapped to the 11 chromosomes (Ch1-Ch11), whereas the NAC-sc gene was located on unassembled scaffold_229. The *arrows* represent the direction of transcription. The position of each gene can be estimated using the scale on the *left*

Figure 1 shows that the 84 common bean NAC genes are distributed across all 11 chromosomes (Ch1-Ch11); however, in the most recently released sequences, Phvul.L010000 remained on as-of-yet unmapped scaffolds. The distributions of common bean NAC genes across the chromosome appeared to be non-random (Fig. 1). Only two NAC genes are distributed on Ch10, the lowest number of genes on a chromosome; on Ch2, 14 NAC genes were identified, the highest number of genes. A number of clusters of NAC genes are evident on the chromosomes, particularly on those with high densities of NAC genes. For example, NAC-Ch9.6 and NAC-Ch9.7 were cluster localized on a 14-kb segment on Ch9, and NAC-Ch5.10 and NAC-Ch5.11, NAC-Ch5.7 and NAC-Ch5.8 are in a cluster on 50-kb and 54-kb fragments of Ch5, respectively. However, NAC-Ch7.3 and NAC-Ch7.4 are arranged in a cluster localized to a 67-kb segment on Ch7 (Fig. 1). In addition, NAC-Ch2.8 and NAC-Ch2.9 are organized in another cluster within a 103-kb fragment on Ch2, whereas NAC-Ch1.5 and NAC-Ch1.6 are arranged in a cluster localized to a 110-kb segment on Ch1 (Fig. 1).

### Putative promoter region analysis of the NAC gene family

TFs bind to the DNA on specific cis-acting regulatory elements (CAREs), which determine the initiation of transcription and are among the most important gene structures [48]. CAREs are short conserved motifs of 5 to 20 nucleotides usually found within the 1500 bp upstream of genes, known as the promoter region [48]. To further investigate transcriptional regulation and the potential functions of NAC subfamily genes in common bean, the promoter regions of the NAC genes (1500-bp sequences upstream of the translational start site) were analyzed using the PlantCARE database to identify putative CAREs. A total of 83 similar CAREs associated with developmental processes, light responsiveness, biotic stress, hormones and other functions were identified in the promoter regions of these NAC genes (Additional file 2: Table S2). All promoters of common bean NAC genes were predicted to contain an essential element, such as a TATA box and a CAAT box. Of these CAREs, several cis-elements related to tissue-specific expression, such as root-specific (AS1 and Motif I), seed-specific (RY element), endosperm-specific (GCN4 and Skn-1 motif), and meristem-specific (dOCT and CCGTACC box) cis-elements, were present in NAC gene promoters. We also observed numerous light-responsive cis-elements widely distributed in the promoter regions of NACs in common bean, such as as-2 box, AE-box, G-box, and GAG-motif. CAREs involved in plant hormones, such as gibberellin-responsive elements (GARE motif and P box), an ethylene-responsive element (ERE), auxin-responsive elements (TGA element and AuxRR core), MeJA-responsive elements (TGACG motif and CGTCA motif) and ABA-responsive elements (ABRE and CE3), were also identified. In particular, important elements in abiotic stress, including heat stress-responsive element (HSE), drought-responsive element (MBS), wound-responsive element (WUN motif), low-temperature element (LTR), cold and dehydration-responsive element (C repeat/DRE) and defense and stress-responsive element (TC-rich repeats) were detected. These results clearly suggest that NAC TFs might respond to abiotic stresses and have potential functions in enhancing abiotic stress resistance. For instance, Phvul.004G029900 and Phvul.005G121800 had up to five types of abiotic stress CAREs. Furthermore, HSE, MBS, WUN-motif, LTR and TC-rich repeats were identified in Phvul.004G029900. HSE, MBS, WUN-motif, C-repeat/DRE and TC-rich repeats were identified in Phvul.005G121800. In addition, Phvul.001G192000, Phvul.002G061000, Phvul.004G075500, Phvul.005G084600, Phvul.007G085600, Phvul.008G189100, Phvul.009G008000 and Phvul.009G039000 had four types of abiotic stress CAREs.

### Phylogenetic relationships, conserved motifs and gene structure analysis of the NAC gene

**Fig. 2:**
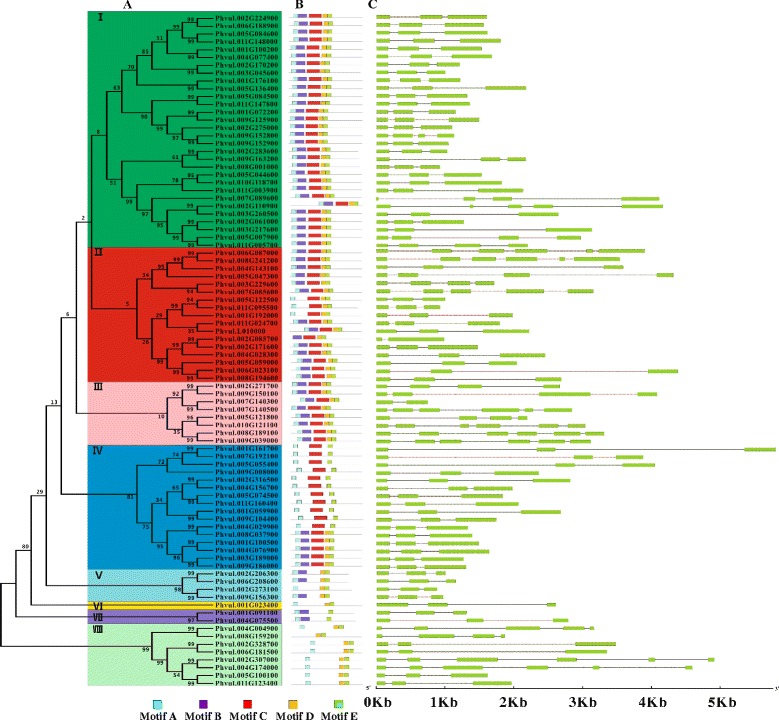
Phylogenetic relationships, gene structure and motif composition of NAC genes in common bean. **a** The phylogenetic tree of NAC genes from common bean was constructed in MEGA4.0 using the Neighbor-Joining (NJ) method with 1,000 bootstrap replicates. **b** The conserved motifs of common bean NAC genes were elucidated by MEME. The conserved motifs are represented by the different colored boxes. The black lines represent the non-conserved sequences. **c** Exon/intron structures of NAC genes from common bean. Exons and introns are represented by green boxes and black lines, respectively. The sizes of exons and introns can be estimated using the scale below

To determine the phylogenetic relationships between NAC genes in common bean, an unrooted phylogenetic tree with 86 complete NAC protein sequences was constructed (Fig. 2a). The phylogenetic tree revealed that NAC family proteins can be classified into eight major groups: I, II, III, IV, V, VI, VII and VIII (Fig. 2a), consistent with previous reports [8, 49]. Group I is the largest clade, with 29 members, and accounts for 33.7 % of all NAC TFs, and groups II and IV contain the same number of members (17). Group VII contains only one member, Phvul.001G023400, and groups I, II, III and IV each contain two subgroups.

**Fig. 3:**
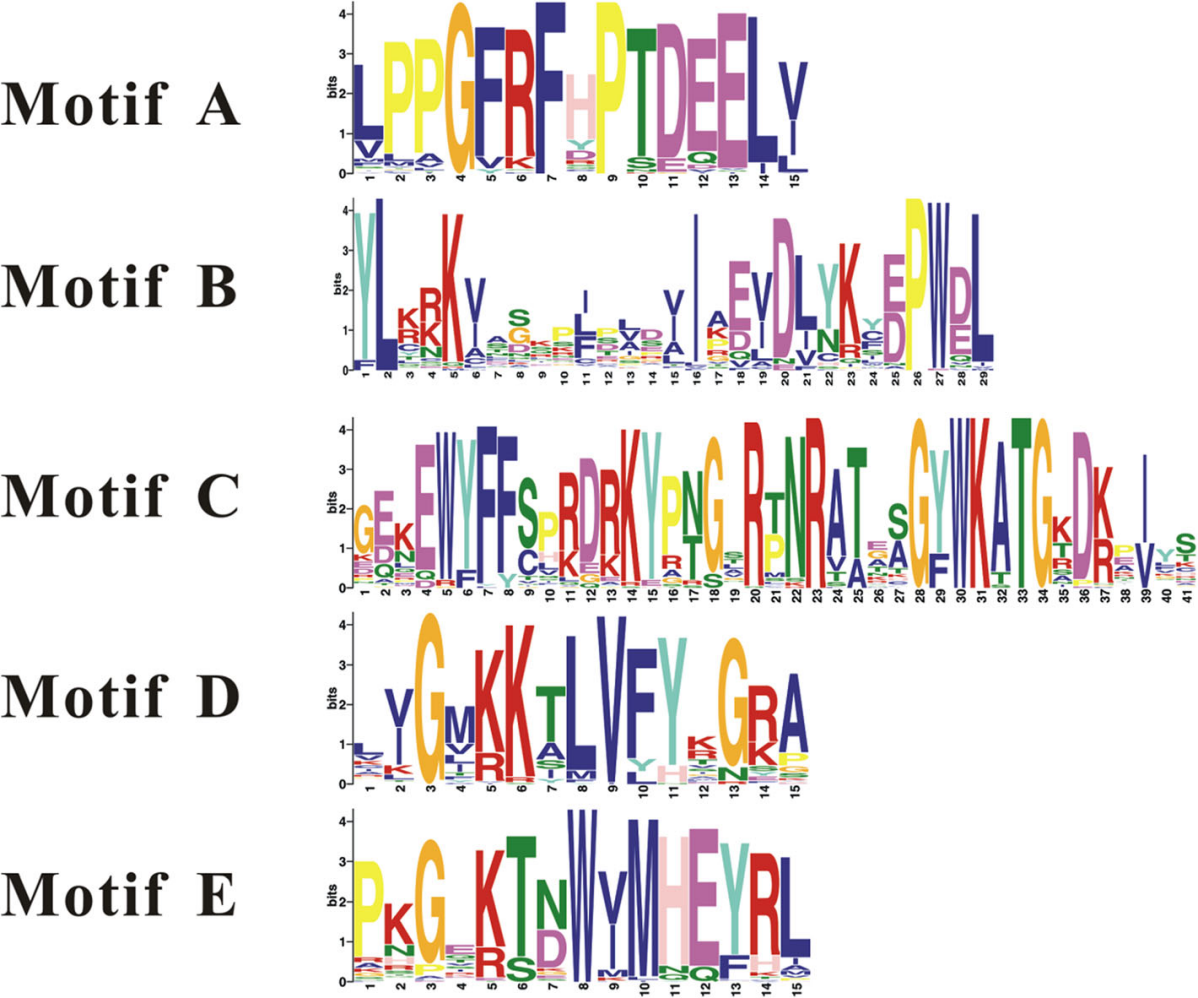
The conserved motifs of common bean NAC genes. The bit score indicates the information content for each position in the sequence

The N-termini of NAC TFs contain five subdomains (A-E) [8]. Thus, we analyzed the conserved motifs of NAC TFs from common bean using the MEME program [50] (Figs. 2b, and 3 and Additional file 3: Table S3). The motif distribution analyses of the NAC proteins revealed that 56 of 86 (65.1 %) common bean NAC proteins contain all five domains, domains A, B, C, D and E (Fig. 2b and Additional file 3: Table S3). Nine (10.5 %) NAC proteins lack one domain (A, B or C); nine (10.5 %) NAC proteins lack domains B and C; eleven (12.8 %) NAC proteins lack B and D; and only one protein, Phvul.008 g159200, lacks three domains (A, B and C). All common bean NAC domains (86) contain motif E, the most highly conserved motif in common bean NACs. Domain A is also relatively highly conserved; only Phvul.002G085700 and Phvul.008 g159200 lack motif A. However, motif B is the least conserved motif in common bean NACs. For instance, all members of groups I and III contain all five motifs (A-E), whereas the members of group VIII (expect for Phvul.008 g159200) contain motifs A, D and E. By contrast, the conserved motif appears to be more variable in groups II, IV, V and VI.

To analyze the structural diversity of NAC genes, we compared the exon/intron organization in the coding sequences of individual NAC genes in common bean using GSDS 2.0. The detailed gene structures are shown in Fig. 2c. Based on the results of gene structure prediction, the number of introns ranges from one to five in the common bean NAC gene family. Among these NAC genes, most NAC genes have two introns, whereas two members have one intron. Overall, genes with highly similar gene structures were clustered in the same phylogenetic group of common bean NAC genes.

### Expression pattern of NAC TFs in common bean

**Fig. 4:**
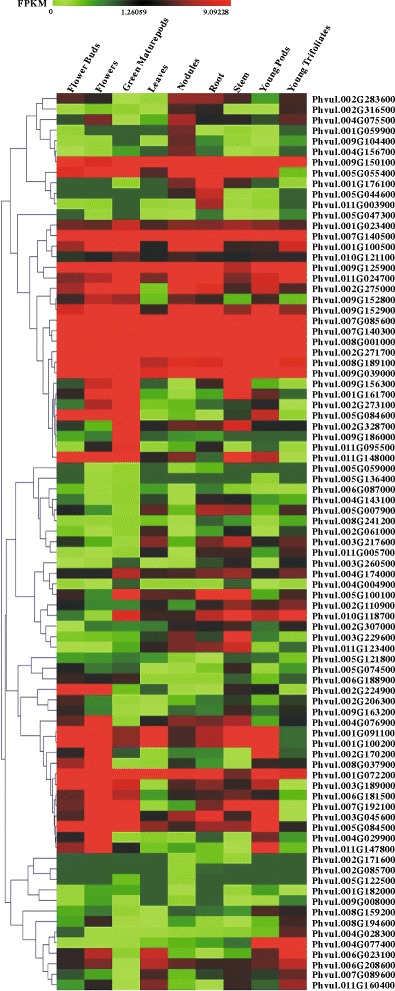
Heat map of expression profiles for NAC genes across different tissues. The expression data were generated from the Phytozome database and viewed in MeV software. Hierarchical clustering was performed for the transcript ratios from all conditions. The color scale shown below represents expression values, with *green* indicating low levels and *red* indicating high levels of transcript abundance

The coding sequences of all NAC domains of common bean were used to search the expression database using Phytozome. Expression data are not available for Phvul.L010000.1, and the expression profiles of 85 NAC genes in 9 common bean tissues, including young trifoliates, leaves, flower buds, flowers, green mature pods, young pods, roots, stems, and nodules, were obtained. No tissue expressed all 85 NAC genes (Additional file 4: Table S4), but the majority of the TFs coexisted in all tissues (62 genes, 72.94 %). NAC TFs were expressed in some tissues but not others. NAC TFs were most abundant in nodules (84 genes, 98.82 %), followed by young pods and roots (80 genes, 94.12 %), flowers (79 genes, 92.94 %), and stems (78 genes, 91.67 %). Few NAC TFs were expressed in the leaves (71 genes, 93.53 %). We constructed an expression profile heat map based on expression data in different organs of NAC TFs (Fig. 4). All NAC TFs with expression profiles were clustered into 6 groups based on their expression patterns. Moreover, five NAC TFs (Phvul.002G271700, Phvul.007G140500, Phvul.007G085600, Phvul.007G140300 and Phvul.008G001000) were highly expressed in all common bean organs. No gene was specifically expressed in only one tissue. Phvul.002G085700 was specifically expressed in nodules and roots, whereas Phvul.005G122500 was specifically expressed in nodules and green mature pods. The other NAC genes were expressed in at least three tissues.

### Expression profiles of NAC TFs under drought stresses

Numerous NAC domain proteins have been implicated in plant drought stress [1–3]. To determine the expression profiles of NAC TFs under drought stress, 86 NAC genes were analyzed using transcriptome and qRT-PCR data. The transcriptome data obtained from our previous report described the expression profiling of the genotypes Long 22-0579 (drought tolerant) and Naihua (drought sensitive) in response to drought stress [51].

**Fig. 5:**
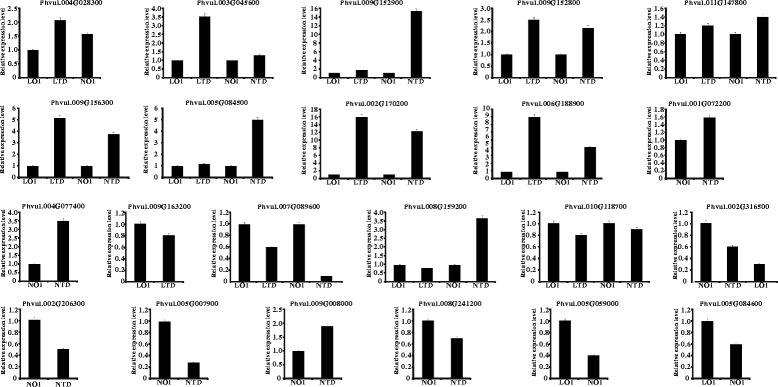
qRT-PCR validation of drought-related NAC proteins from common bean

We detected 13 differentially expressed NAC genes (DENs) between samples LOI and LTD and 18 genes between NOI and NTD. In this study, ‘up-regulated’ and ‘down-regulated’ were denoted in accordance with the results from a previous study (Table 1). Between samples LOI and LTD, more DENs were up-regulated (9) than down-regulated (4). Similarly, more DENs were up-regulated (10) than down-regulated (8) between NOI and NTD. Among these DENs, eleven NAC genes shared a common expression pattern in Long 22-0579 or Naihua under drought stress. Two genes (Phvul.004G028300 and Phvul.009G163200) were up- or down-regulated under drought stress only in the drought-tolerant genotype, whereas five genes were differentially expressed under drought stress only in the drought-sensitive genotype. In addition, four genes (Phvul.002G3616500, Phvul.004G028300, Phvul.005G05900 and Phvul.005G084600) exhibited differential expression under drought stress between different cultivars (Long 22-0579 or Naihua). However, Phvul.002G316500 and Phvul004G028300 were also differentially expressed under drought stress in the drought-sensitive and drought-tolerant genotypes, respectively. All candidate DENs obtained by RNA-seq analysis were further validated by RT-PCR (Fig. 5 and Additional file 5: Table S5). The expression profiles of 20 candidates, excluding Phvul.008G159200 and Phvul.009G008000, were generally in agreement with the predictions from the RNA-seq results (Additional file 6: Table S6). These results suggest that these DENs are related to drought stress.

**Table 1:** Selected differentially expressed NAC proteins between different treatment and cultivars

Expression pattern		Genes	Fold change (LOI to LTD)	Fold change (NOI to NTD)	Fold change (LOI to NOI)
Up-regulated	LOI/LTD	Phvul.004G028300	3.41		3.80
	LOI/NOI and NOI/NTD	Phvul.003G045600	2.78	2.74	
		Phvul.009G152900	3.16	3.04	
		Phvul.009G152800	3.53	3.13	
		Phvul.011G147800	4.28	4.46	
		Phvul.009G156300	5.03	5.11	
		Phvul.005G084500	5.56	5.52	
		Phvul.002G170200	3.13	5.58	
		Phvul.006G188900	5.44	5.86	
	NOI/NTD	Phvul.001G072200		2.37	
		Phvul.004G077400		3.31	
Down-regulated	LOI/LTD	Phvul.009G163200	−3.69		
	LOI/NOI and NOI/NTD	Phvul.007G089600	−2.72	−5.45	
		Phvul.008G159200	−2.67	−4.86	
		Phvul.010G118700	−2.38	−2.98	
	NOI/NTD	Phvul.002G316500		−4.18	3.14
		Phvul.002G206300		−4.11	
		Phvul.005G007900		−3.74	
		Phvul.009G008000		−3.39	
		Phvul.008G241200		−2.38	
	LOI/NOI	Phvul.005G059000			−3.52
		Phvul.005G084600			−2.15

In general, orthologous genes of different plants usually have similar functions [52]. Thus, common bean NAC genes may have functions similar to those of genes in the same subgroup with known functions. We built a phylogenetic tree based on the amino acid sequences of NAC proteins from common bean and known drought-related NAC proteins from other species, including rice, *Arabidopsis*, soybean, chickpea, and wheat (Additional file 7: Figure S1). A total of 20 DENs belonged to different subgroups including drought-related NAC genes. These results indicate that orthologs such as Phvul.009G15280, Phvul.005G 084500 and other DENs may have similar functions and that these DENs may be associated with drought stress. However, we also observed that Phvul.005G059000 and Phvul.004G028300 belonged to the same subgroup without any known-function NAC genes. Furthermore, MBS is a cis-acting regulatory element that is predicted to serve as an MYB binding site involved in drought inducibility. *TsApx6* (*Thellungiella salsuginea*) is involved in the response to drought stress and contains an MBS element in its promoter [53]. Among these related NAC genes of common bean, 16 genes contain MBS cis-elements (e.g., Phvul.003G045600, Phvul.011G147800 and Phvul.009G156300). These results support the involvement of these NAC genes in drought resistance. We also compared the cis-acting regulatory elements and the promoters of DENs and orthologues from different plants (soybean, rice, and *Arabidopsis*) (Additional file 8: Table S7). Among these CAREs, in addition to essential elements and enhancers, we found 18 conservative CAREs (more than half of the genes) in drought-responsive genes (e.g., ARE, circadian, HSE, MBS, Skn-1_motif, CGTCA, and TGACG). Among these CAREs, MBS involves in drought inducibility, and CGTCA and TGACG involve in MeJA responsiveness. These conservative CAREs maybe play an important role in regulating drought resistance.

## Discussion

Common bean is a food legume. The seeds of common bean are an important food source, and common bean plants also contribute to soil fertility. Whole-genome sequences of many food legumes, including pigeonpea [17], chickpea [18, 19], mung bean [22], and adzuki bean [23], have recently been released. The genome of common bean was completed with two *P. vulgaris* accessions: an Andean genotype (*Phaseolus vulgaris* L., G19833) and a Mesoamerican genotype (*Phaseolus vulgaris* L., BAT93) [20, 21]. These sequence data provide rich resources for comparative genomic analyses and genome and gene evolution studies. The NAC protein family is one of the largest families of TFs and is involved in plant development and response to abiotic and biotic stresses. NAC proteins have been studied in many plants, including maize, soybean, *Oryza sativa*, *Arabidopsis thaliana*, and *Opulous trichocarpa* [8, 54–56], but this study is the first to identify and characterize NAC proteins encoded in the common bean genome.

In this study, we analyzed 86 non-redundant NAC genes from common bean, fewer NAC genes than in other grasses, for example, 163 in *Populus* [54], 105 in *Arabidopsis* [8], 140 in rice [55], and 101 in soybean [56]. We also analyzed the gene structures and conserved motifs of the NAC TFs. The common bean NAC genes contained one to five introns. The exon/intron numbers of common bean NAC genes differ from those of other plants, such as *Populus*, which has a range of zero to eight. However, the number of conserved motifs in common bean NAC genes was similar to that of other species, including *Populus*, rice, soybean and *Arabidopsis*. However, the diversity of gene structures and conserved motifs may also indicate that common bean NACs are functionally diversified, with roles in shoot apical meristem development, floral morphogenesis, lateral root development, leaf senescence, embryo development, cell cycle control, hormone signaling, abiotic stresses and defense responses. In general, proteins with similar sequences have similar functions, and we therefore analyzed the functions of common bean NAC TFs based on the phylogenetic tree of NAC proteins. Phvul.005G074500 and Phvul.011G160400 may be involved in shoot apical meristem formation and development because they clustered into one subgroup with CUC1 and CUC2 [57, 58]. Moreover, ATAF1, ATAF2, Phvul.009G125900, Phvul.001G072200, Phvul.002G275000, Phvul.009G152800 and Phvul.009G152900 clustered into one group and may be involved in wounding [59, 60]. Phvul.007G089600 and VND 7 clustered into one subgroup and have been proposed as regulators of vascular vessel formation [14]. Some genes may participate in responses to abiotic stress, such as Phvul.011G147800, GmNAC3, GmNAC4, ANAC019, ANAC055 and ANAC072 under salt stress [61, 62]. Some genes (ANAC053 and Phvul.007G140500) have been reported to be mostly involved in heat response [63] but may have more functions; for example, Phvul.001G072200, Phvul.009G125900 and OsNAC6 are involved in the response to abiotic stresses, such as high salinity, ABA treatment and cold [64]. The functions of many NAC family genes remain unknown. Future studies will focus on discovering novel functions of NAC genes, particularly of genes specific to common bean.

In this paper, we focused on the function of NAC genes under drought stress. In the present study, we identified 22 common bean NAC TFs that were induced by drought stresses based on transcriptome data; these genes were of two types: differentially expressed between drought-tolerant/sensitive genotypes and differentially expressed between treatment/control. Furthermore, quantitative real-time PCR demonstrated that the expression profiles of the 20 candidates were generally in agreement with the predictions from the RNA-seq results, indicating that these genes are functionally associated with the drought-stress response. In addition, the phylogenetic tree of common bean NAC genes and known-function NAC genes from other species also suggested that these 22 NAC genes may be related to drought stress. For example, one group included five common bean NAC genes and 14 known-function NAC genes that are all induced by drought stress [1, 39, 40, 42, 44, 65–70]. The members of this subfamily are also the most widely studied and play important roles in the NAC family. Another group included five common bean NAC genes and CarNAC3 from chickpea [44], MsNAC from *Medicago sativa* [71], StNAC2 from potato [72], ZMNAC111 from maize [73], ANAC002 and ANAC047 from *Arabidopsis* [31, 74] and OsNAC10 from rice [43], all of which are induced by drought. Phvul.005G059000 and Phvul.004G028300 belong to a group without any drought-related NAC proteins. These results suggest that Phvul.005G059000 and Phvul.004G028300 may be a new class of NAC TFs that are not involved in drought resistance.

## Conclusions

We comprehensively identified NAC genes in common bean based on the genome sequence. This study identified a non-redundant set of 86 NAC genes in common bean. Detailed analyses identified phylogenetic relationships, conserved motifs, gene structure and expression profiles of common bean NAC genes. Our research provides useful information for further research on the function of NAC in common bean and will accelerate functional genomics studies and molecular breeding programs. Moreover, the candidate drought-responsive NAC genes identified in common bean will provide a new resource for molecular breeding in food legumes and other crops.

## Methods

### Searching for NAC family members in common bean

Whole-genome sequences of common bean were downloaded from the Phytozome genome database [19]. The hidden Markov model (HMM) profile of the NAC family (PF02365) was extracted from the Pfam database [75], and the NAC HMM profile was used to search the common bean whole-genome protein database for target hits with the NAC domain by HMMER3.0 [76]. Based on the sequence ID of the NAC protein, the coding sequences and genome sequences were extracted from the common bean whole genome sequence database. Transcriptome data of the genotypes Long 22-0579 (drought tolerant) and Naihua (drought sensitive) were downloaded from NCBI (GenBank accession no.: bean LTD SAMN03223377, bean NOI SAMN03223381, bean NTD SAMN03223380, and bean LOI SAMN03223378).

### Data analyses

ExPASy was used to determine the number of amino acids in the open reading frame (ORF), molecular weight (MW), isoelectric point (pI) and length of the open reading frame (length) of each gene (http://www.expasy.ch/tools/pi_tool.html). Subcellular localization was predicted using Softberry (http://linux1.softberry.com/). MEGA4.0 was also used to generate neighbor-joining (NJ) trees with bootstrap values. The exon/intron organization of each NAC gene was visualized in the Gene Structure Display Server program [77]. Motifs of the NAC proteins were displayed using MEME [50]. The upstream promoter sequences of NAC genes were identified using the PlantCARE database [78]. The heat map was viewed in the MeV tool (http://www.tm4.org/mev.html). The upstream promoter sequences of NAC genes from rice, soybean and *Arabidopsis* were downloaded from the Phytozome database.

### Expression pattern analysis and qRT-PCR analysis

Transcript data were obtained from the Phytozome database for young trifoliates, leaves, flower buds, flowers, green mature pods, young pods, roots, stems, and nodules (https://phytozome.jgi.doe.gov/phytomine/template.do?name=One_Gene_Expression&scope=global).

Total RNA was extracted from leaves using TRIzol reagent according to the manufacturer’s instructions (Tiangen, Beijing, China), and first-strand cDNA was synthesized using the SuperScript II reverse transcriptase kit (Invitrogen). Real-time PCR was performed on an ABI PRISM 7300 Sequence Detection System (Applied Biosystems) using SYBR Premix Ex Taq (TAKARA). Relative expression levels were calculated using the 2^-△△CT^ method. qRT-PCR was conducted using the common bean actin gene (GenBank accession no.: EU369188.1) as the control. Specific primers for qRT-PCR were designed using primer 5.0 (http://www.premierbiosoft.com/primerdesign/).

The common bean cultivars Long 22-0579 (drought-tolerant genotype) and Naihua (drought-sensitive genotype) were employed to identify genes involved in drought stress using RNA-seq. Seedlings of the cultivars were grown in plastic pots (23 cm × 18 cm × 18 cm) under a 14/10 h photoperiod at 25 °C (day) and 20 °C (night) in a greenhouse (China, Beijing, 116°46′E, 39°92′N). The water content was measured three times a week, and any water lost was replaced in the pots to maintain equivalent levels according to the treatment requirements. Twenty-five plants were used in each treatment. All plants were irrigated to field capacity until 4 weeks after seeding. For the terminal drought treatment, watering was restricted to 25 % of the field capacity beginning 5 weeks after seeding. For optimal irrigation, the pots were maintained at the field capacity throughout the experiment [49].

The method employed for the identification of differentially expressed NAC genes (DENs) from transcriptome data involved tests implemented using DEGseq, and the corresponding significance thresholds applied were determined using the likelihood ratio test, Fisher’s exact test, the MA-plot-based method with a random sampling model (*p*-value ≤ 0.001) and the fold-change threshold of MA-plot log_2_ normalized fold changes ≥2 [49].

## Additional files

Additional file 1: Table S1. The 106 putative members of the NAC family of genes identified in common bean. (XLSX 67 kb) Additional file 2: Table S2. Predicted promoter elements of common bean NAC genes. (XLSX 65 kb) Additional file 3: Table S3. Conserved motifs of common bean NAC domain proteins. (XLSX 11 kb) Additional file 4: Table S4. Expression data of NAC genes in common bean. (XLSX 38 kb) Additional file 5: Table S5. The primer sequences for DENs used for qRT-PCR. (XLSX 22 kb) Additional file 6: Table S6. Transcriptome data (RPKM) of DENs. (XLSX 11 kb) Additional file 7: Figure S1. Phylogenetic relationship of common bean NAC genes with drought-related NAC genes from other species. The phylogenetic tree was constructed in MEGA6.0 using the neighbor-joining method with 1000 bootstrap replicates. Each common bean drought-related NAC protein is indicated by a red dot. Sequences of drought-related NAC genes from other species downloaded from NCBI: CarNAC1(ACA96935), CarNAC2(ACA96936), CarNAC3(FJ356671), CarNAC4(ACS94037), CarNAC5(ACS94038), CarNAC6(ACS94039), CcNAC1(AHJ38168), CcNAC2(AHJ38169), SNAC2(XP_015620920), SNAC3(XP_015615070), ONAC022(AK107090), ONAC045(AK067922), OsNAC6(EAY76735), OsNAC10(XP_015645677), OsNAC44(AIQ84824), OsNAC52(AAT44250), OMTN2(XP_015633922), OMTN3(XP_015620576), OMTN4(XP_015643121), OMTN6(XP_015648318), TaNAC2(AAU08786), TaNAC47(KT345698), TaNAC67(KF646593), HvsNAC1(JF796130), ZmNAC55(AFW67212), ZmNAC111(NP_001183815), ZmSNAC1(AEY78612), SbSNAC1(AGG40203), MsNAC(HM237304), StNAC2(NP_001275015), ANAC002(At1g01720), ANAC016(At1g34180), ANAC019(At1g52890), ANAC030(At1g71930), ANAC047(At3g04070), ANAC055(At3g15500), ANAC069(At4g01550), ANAC072(At4g27410), ANAC087(At5g18270), ANAC096(At5g46590). (TIF 3509 kb) Additional file 8: Table S7. Predicted promoter elements of NAC genes from common bean, rice, soybean and Arabidopsis. (XLSX 199 kb)

## Abbreviations

CAREs: Cis-acting regulatory elements
CDS: Coding sequence
DENs: Differentially expressed NAC genes
HMM: Hidden Markov model
LOI: NOI, LTD, NTD, cultivars (Long 22-0579 or Naihua) and the treatments (optimal irrigation or terminal drought) applied to their sampling source
MW: Molecular weight
NAC: NAM, ATAF1/2 and CUC2
NJ: Neighbor-joining
ORF: Open reading frame
pI: Isoelectric point
qRT-PCR: quantitative real-time PCR
TFs: Transcription factors

## References

[1] Zhang L, Zhang L, Xia C, Zhao G, Jia J, Kong X (2016). The novel wheat transcription factor TaNAC47 enhances multiple abiotic stress tolerances in transgenic Plants. Front Plant Sci.

[2] Fang Y, Liao K, Du H, Xu Y, Song H, Li X, Xiong L (2015). A stress-responsive NAC transcription factor SNAC3 confers heat and drought tolerance through modulation of reactive oxygen species in rice. J Exp Bot.

[3] Hong Y, Zhang H, Huang L, Li D, Song F (2016). Overexpression of a stress-responsive NAC transcription factor Gene ONAC022 improves drought and Salt tolerance in Rice. Front Plant Sci.

[4] Jin C, Huang XS, Li KQ, Yin H, Li LT, Yao ZH, Zhang SL (2016). Overexpression of a bHLH1 transcription factor of Pyrus ussuriensis confers enhanced Cold tolerance and increases expression of stress-responsive genes. Front Plant Sci.

[5] He Q, Jones DC, Li W, Xie F, Ma J, Sun R, Wang Q, Zhu S, Zhang B (2016). Genome-wide identification of R2R3-MYB genes and expression analyses during abiotic stress in Gossypium raimondii. Sci Rep.

[6] Jin J, Zhang H, Kong L, Gao G, Luo J (2014). PlantTFDB 3.0: a portal for the functional and evolutionary study of plant transcription factors. Nucleic Acids Res.

[7] Schmutz J, Cannon SB, Schlueter J, Ma J, Mitros T, Nelson W, Hyten DL, Song Q, Thelen JJ, Cheng J, Xu D, Hellsten U, May GD, Yu Y, Sakurai T, Umezawa T, Bhattacharyya MK, Sandhu D, Valliyodan B, Lindquist E (2010). Genome sequence of the palaeopolyploid soybean. Nature.

[8] Ooka H, Satoh K, Doi K, Nagata T, Otomo Y, Murakami K, Matsubara K, Osato N, Kawai J, Carninci P, Hayashizaki Y, Suzuki K, Kojima K, Takahara Y, Yamamoto K, Kikuchi S (2003). Comprehensive analysis of NAC family genes in Oryza sativa and Arabidopsis thaliana. DNA Res.

[9] Aida M, Ishida T, Fukaki H, Fujisawa H, Tasaka M (1997). Genes involved in organ separation in Arabidopsis: an analysis of the cup-shaped cotyledon mutant. Plant Cell.

[10] Kikuchi K, Ueguchi-Tanaka M, Yoshida KT, Nagato Y, Matsusoka M, Hirano HY (2000). Molecular analysis of the NAC gene family in rice. Mol Gen Genet.

[11] Xie Q, Frugis G, Colgan D, Chua NH (2000). Arabidopsis NAC1 transduces auxin signal downstream of TIR1 to promote lateral root development. Genes Dev.

[12] Ko JH, Yang SH, Park AH, Lerouxel O, Han KH (2007). ANAC012, a member of the plant-specific NAC transcription factor family, negatively regulates xylary fiber development in Arabidopsis thaliana. Plant J.

[13] Zhong R, Lee C, Ye ZH (2010). Functional characterization of Poplar Wood-associated NAC domain transcription factors. Plant Physiol.

[14] Yamaguchi M, Kubo M, Fukuda H, Demura T (2008). Vascular-related NAC-DOMAIN7 is involved in the differentiation of all types of xylem vessels in Arabidopsis roots and shoots. Plant J.

[15] Taoka K, Yanagimoto Y, Daimon Y, Hibara K, Aida M, Tasaka M (2004). The NAC domain mediates functional specificity of CUP-SHAPED COTYLEDON proteins. Plant J.

[16] Maugarny-Calès A, Gonçalves B, Jouannic S, Melkonian M., Wong G.K.-S., Laufs P. Apparition of the NAC transcription factors predates the emergence of land plants. Mol Plant. 2016, in press. doi: 10.1016/j.molp.2016.05.016. 10.1016/j.molp.2016.05.016 27302340

[17] Varshney RK, Chen W, Li Y, Bharti AK, Saxena RK, Schlueter JA, Donoghue MT, Azam S, Fan G, Whaley AM, Farmer AD, Sheridan J, Iwata A, Tuteja R, Penmetsa RV, Wu W, Upadhyaya HD, Yang SP, Shah T, Saxena KB, Michael T, McCombie WR, Yang B, Zhang G, Yang H, Wang J, Spillane C, Cook DR, May GD, Xu X, Jackson SA (2012). Draft genome sequence of pigeonpea (Cajanus cajan), an orphan legume crop of resource-poor farmers. Nat Biotechnol.

[18] Jain M, Misra G, Patel RK, Priya P, Jhanwar S, Khan AW, Shah N, Singh VK, Garg R, Jeena G, Yadav M, Kant C, Sharma P, Yadav G, Bhatia S, Tyagi AK, Chattopadhyay D (2013). A draft genome sequence of the pulse crop chickpea (Cicer arietinum L.). Plant J.

[19] Varshney RK, Song C, Saxena RK, Azam S, Yu S, Sharpe AG, Cannon S, Baek J, Rosen BD, Tar'an B, Millan T, Zhang X, Ramsay LD, Iwata A, Wang Y, Nelson W, Farmer AD, Gaur PM, Soderlund C, Penmetsa RV, Xu C, Bharti AK, He W, Winter P, Zhao S, Hane JK, Carrasquilla-Garcia N, Condie JA, Upadhyaya HD, Luo MC, Thudi M, Gowda CL, Singh NP, Lichtenzveig J, Gali KK, Rubio J, Nadarajan N, Dolezel J, Bansal KC, Xu X, Edwards D, Zhang G, Kahl G, Gil J, Singh KB, Datta SK, Jackson SA, Wang J, Cook DR (2013). A draft genome sequence of the pulse crop chickpea (Cicer arietinum L.). Nat Biotechnol.

[20] Schmutz J, McClean PE, Mamidi S, Wu GA, Cannon SB, Grimwood J, Jenkins J, Shu S, Song Q, Chavarro C, Torres-Torres M, Geffroy V, Moghaddam SM, Gao D, Abernathy B, Barry K, Blair M, Brick MA, Chovatia M, Gepts P, Goodstein DM, Gonzales M, Hellsten U, Hyten DL, Jia G, Kelly JD, Kudrna D, Lee R, Richard MM, Miklas PN, Osorno JM, Rodrigues J, Thareau V, Urrea CA, Wang M, Yu Y, Zhang M, Wing RA, Cregan PB, Rokhsar DS, Jackson SA (2014). A reference genome for common bean and genome-wide analysis of dual domestications. Nat Genet.

[21] Vlasova A, Capella-Gutiérrez S, Rendón-Anaya M, Hernández-Oñate M, Minoche AE, Erb I, Câmara F, Prieto-Barja P, Corvelo A, Sanseverino W, Westergaard G, Dohm JC, Pappas GJ, Saburido-Alvarez S, Kedra D, Gonzalez I, Cozzuto L, Gómez-Garrido J, Aguilar-Morón MA, Andreu N, Aguilar OM, Garcia-Mas J, Zehnsdorf M, Vázquez MP, Delgado-Salinas A, Delaye L, Lowy E, Mentaberry A, Vianello-Brondani RP, García JL, Alioto T, Sánchez F, Himmelbauer H, Santalla M, Notredame C, Gabaldón T, Herrera-Estrella A, Guigó R (2016). Genome and transcriptome analysis of the Mesoamerican common bean and the role of gene duplications in establishing tissue and temporal specialization of genes. Genome Biol.

[22] Kang YJ, Kim SK, Kim MY, Lestari P, Kim KH, Ha BK, Jun TH, Hwang WJ, Lee T, Lee J, Shim S, Yoon MY, Jang YE, Han KS, Taeprayoon P, Yoon N, Somta P, Tanya P, Kim KS, Gwag JG, Moon JK, Lee YH, Park BS, Bombarely A, Doyle JJ, Jackson SA, Schafleitner R, Srinives P, Varshney RK, Lee SH (2014). Genome sequence of mung bean and insights into evolution within Vigna species. Nat Commun.

[23] Yang K, Tian Z, Chen C, Luo L, Zhao B, Wang Z, Yu L, Li Y, Sun Y, Li W, Chen Y, Li Y, Zhang Y, Ai D, Zhao J, Shang C, Ma Y, Wu B, Wang M, Gao L (2015). Genome sequencing of adzuki bean (Vigna angularis) provides insight into high starch and low fat accumulation and domestication. Proc Natl Acad Sci U S A.

[24] Sablowski RW, Meyerowitz EM (1998). A homolog of NO APICAL MERISTEM is an immediate target of the floral homeotic genes APETALA3/PISTILLATA. Cell.

[25] Wang F, Lin R, Feng J, Chen W, Qiu D, Xu S (2015). TaNAC1 acts as a negative regulator of stripe rust resistance in wheat, enhances susceptibility to Pseudomonas syringae, and promotes lateral root development in transgenic Arabidopsis thaliana. Front Plant Sci.

[26] Kim HJ, Nam HG, Lim PO (2016). Regulatory network of NAC transcription factors in leaf senescence. Curr Opin Plant Biol.

[27] Chen Y, Qiu K, Kuai B, Ding Y (2011). Identification of an nap-like transcription factor BeNAC1 regulating leaf senescence in bamboo (Bambusa emeiensis'Viridiflavus'). Physiol Plant.

[28] Kim SG, Kim SY, Park CM (2007). A membrane-associated NAC transcription factor regulates salt-responsive flowering via FLOWERING LOCUS T in Arabidopsis. Planta.

[29] Park J, Kim YS, Kim SG, Jung JH, Woo JC, Park CM (2011). Integration of auxin and salt signals by the NAC transcription factor NTM2 during seed germination in Arabidopsis. Plant Physiol.

[30] Zhao Y, Sun J, Xu P, Zhang R, Li L (2014). Intron-mediated alternative splicing of Wood-ASSOCIATED NACTRANSCRIPTIONFACTOR1B regulates cell wall thickening during fiber development in populus species. Plant Physiol.

[31] Yu X, Liu Y, Wang S, Tao Y, Wang Z, Shu Y, Peng H, Mijiti A, Wang Z, Zhang H, Ma H (2016). CarNAC4, a NAC-type chickpea transcription factor conferring enhanced drought and salt stress tolerances in Arabidopsis. Plant Cell Rep.

[32] Zhao X, Yang X, Pei S, He G, Wang X, Tang Q, Jia C, Lu Y, Hu R, Zhou G (2016). The miscanthus NAC transcription factor MlNAC9 enhances abiotic stress tolerance in transgenic Arabidopsis. Gene.

[33] Chen SP, Lin IW, Chen X, Huang YH, Chang HC, Lo HS, Lu HH, Yeh KW. Sweet potato NAC transcription factor, IbNAC1, up-regulates sporamin gene expression by binding the SWRE motif against mechanical wounding and herbivore attack. Plant J. 2016;21. doi: 10.1111/tpj.13171 10.1111/tpj.13171 26996980

[34] Guo WL, Wang SB, Chen RG, Chen BH, Du XH, Yin YX, Gong ZH, Zhang YY (2015). Characterization and expression profile of CaNAC2 pepper gene. Front Plant Sci.

[35] Yang X, Wang X, Ji L, Yi Z, Fu C, Ran J, Hu R, Zhou G (2015). Overexpression of a miscanthus lutarioriparius NAC gene MlNAC5 confers enhanced drought and cold tolerance in Arabidopsis. Plant Cell Rep.

[36] Lu PL, Chen NZ, An R, Su Z, Qi BS, Ren F, Chen J, Wang XC (2007). A novel drought-inducible gene, ATAF1, encodes a NAC family protein that negatively regulates the expression of stress-responsive genes in Arabidopsis. Plant Mol Biol.

[37] Fujita M, Fujita Y, Maruyama K, Seki M, Hiratsu K, Ohme-Takagi M, Tran LS, Yamaguchi-Shinozaki K, Shinozaki K (2004). A dehydration-induced NAC protein, RD26, is involved in a novel ABA-dependent stress-signaling pathway. Plant J.

[38] Huang Q, Wang Y, Li B, Chang J, Chen M, Li K, Yang G, He G (2015). TaNAC29, a NAC transcription factor from wheat, enhances salt and drought tolerance in transgenic Arabidopsis. BMC Plant Biol.

[39] Mao X, Chen S, Li A, Zhai C, Jing R (2014). Novel NAC transcription factor TaNAC67 confers enhanced multi-abiotic stress tolerances in Arabidopsis. PLoS ONE.

[40] Mao X, Zhang H, Qian X, Li A, Zhao G, Jing R (2012). TaNAC2, a NAC-type wheat transcription factor conferring enhanced multiple abiotic stress tolerances in Arabidopsis. J Exp Bot.

[41] Zheng X, Chen B, Lu G, Han B (2009). Overexpression of a NAC transcription factor enhances rice drought and salt tolerance. Biochem Biophys Res Commun.

[42] Nakashima K, Tran LS, Van Nguyen D, Fujita M, Maruyama K, Todaka D, Ito Y, Hayashi N, Shinozaki K, Yamaguchi-Shinozaki K (2007). Functional analysis of a NAC-type transcription factor OsNAC6 involved in abiotic and biotic stress-responsive gene expression in rice. Plant J.

[43] Jeong JS, Kim YS, Baek KH, Jung H, Ha SH, Do Choi Y, Kim M, Reuzeau C, Kim JK (2010). Root-specific expression of OsNAC10 improves drought tolerance and grain yield in rice under field drought conditions. Plant Physiol.

[44] Peng H, Cheng H, Chen C, Yu X, Yang J, Gao W, Shi Q, Zhang H, Li J, Ma H (2009). A NAC transcription factor gene of chickpea (Cicer arietinum), CarNAC3, is involved in drought stress response and various developmental processes. J Plant Physiol.

[45] Peng H, Cheng HY, Yu XW, Shi QH, Zhang H, Li JG, Ma H (2009). Characterization of a chickpea (Cicer arietinum L.) NAC family gene, CarNAC5, which is both developmentally- and stress-regulated. Plant Physiol Biochem.

[46] Tran LS, Quach TN, Guttikonda SK, Aldrich DL, Kumar R, Neelakandan A, Valliyodan B, Nguyen HT (2009). Molecular characterization of stress-inducible GmNAC genes in soybean. Mol Genet Genomics.

[47] Thu NB, Hoang XL, Doan H, Nguyen TH, Bui D, Thao NP, Tran LS (2014). Differential expression analysis of a subset of GmNAC genes in shoots of two contrasting drought-responsive soybean cultivars DT51 and MTD720 under normal and drought conditions. Mol Biol Rep.

[48] Rombauts S, Florquin K, Lescot M, Marchal K, Rouzé P, van de Peer Y (2003). Computational approaches to identify promoters and cis-regulatory elements in plant genomes. Plant Physiol.

[49] Shen H, Yin Y, Chen F, Xu Y, Dixon RA (2009). A bioinformatic analysis of NAC genes for plant cell wall development in relation to lignocellulosic bioenergy production. Bioenerg Res.

[50] Bailey TL, Boden M, Buske FA, Frith M, Grant CE, Clementi L, Ren J, Li WW, Noble WS (2009). MEME SUITE: tools for motif discovery and searching. Nucleic Acids Res.

[51] Wu J, Wang L, Li L, Wang S (2014). *De Novo* assembly of the common bean transcriptome using short reads for the discovery of drought-responsive genes. PLoS ONE.

[52] Li WH, Yang J, Gu X (2005). Expression divergence between duplicate genes. Trends Genet.

[53] Li Z, Zhang J, Li J, Li H, Zhang G (2016). The functional and regulatory mechanisms of the *thellungiella salsuginea* ascorbate peroxidase 6 (TsAPX6) in response to salinity and water deficit stresses. PLoS ONE.

[54] Hu R, Qi G, Kong Y, Kong D, Gao Q, Zhou G (2010). Comprehensive analysis of NAC domain transcription factor Gene family in populus trichocarpa. BMC Plant Biol.

[55] Fang Y, You J, Xie K, Xie W, Xiong L (2008). Systematic sequence analysis and identification of tissue-specific or stress-responsive genes of NAC transcription factor family in rice. Mol Genet Genomics.

[56] Pinheiro GL, Marques CS, Costa MD, Reis PA, Alves MS, Carvalho CM, Fietto LG, Fontes EP (2009). Complete inventory of soybean NAC transcription factors: sequence conservation and expression analysis uncover their distinct roles in stress response. Gene.

[57] Hibara K, Takada S, Tasaka M (2003). CUC1 gene activates the expression of SAM-related genes to induce adventitious shoot formation. Plant J.

[58] Nikovics K, Blein T, Peaucelle A, Ishida T, Morin H, Aida M, Laufs P (2006). The balance between the MIR164A and CUC2 genes controls leaf margin serration in Arabidopsis. Plant Cell.

[59] Collinge M, Boller T (2001). Differential induction of two potato genes, Stprx2 and StNAC, in response to infection by phytophthora infestans and to wounding. Plant Mol Biol.

[60] Delessert C, Wilson IW, Van Der Straeten D, Dennis ES, Dolferus R (2004). Spatial and temporal analysis of the local response to wounding in Arabidopsis leaves. Plant Mol Biol.

[61] Tran LS, Nakashima K, Sakuma Y, Simpson SD, Fujita Y, Maruyama K, Fujita M, Seki M, Shinozaki K, Yamaguchi-Shinozaki K (2004). Isolation and functional analysis of Arabidopsis stress-inducible NAC transcription factors that bind to a drought-responsive cis-element in the early responsive to dehydration stress 1 promoter. Plant Cell.

[62] Mahajan S, Tuteja N (2005). Cold, salinity and drought stresses: an overview. Arch Biochem Biophys.

[63] Lee S, Lee HJ, Huh SU, Paek KH, Ha JH, Park CM (2014). The Arabidopsis NAC transcription factor NTL4 participates in a positive feedback loop that induces programmed cell death under heat stress conditions. Plant Sci.

[64] Hu H, You J, Fang Y, Zhu X, Qi Z, Xiong L (2008). Characterization of transcription factor gene SNAC2 conferring cold and salt tolerance in rice. Plant Mol Biol.

[65] Mao H, Yu L, Han R, Li Z, Liu H (2016). ZmNAC55, a maize stress-responsive NAC transcription factor, confers drought resistance in transgenic Arabidopsis. Plant Physiol Biochem.

[66] Gao F, Xiong A, Peng R, Jin X, Xu J, Zhu B, Chen J, Yao Q (2010). OsNAC52, a rice NAC transcription factor, potentially responds to ABA and confers drought tolerance in transgenic plants. Plant Cell Tiss Organ Cult.

[67] McGrann GR, Steed A, Burt C, Goddard R, Lachaux C, Bansal A, Corbitt M, Gorniak K, Nicholson P, Brown JK (2015). Contribution of the drought tolerance-related stress-responsive NAC1 transcription factor to resistance of barley to Ramularia leaf spot. Mol Plant Pathol.

[68] Lu M, Ying S, Zhang DF, Shi YS, Song YC, Wang TY, Li Y (2012). A maize stress-responsive NAC transcription factor, ZmSNAC1, confers enhanced tolerance to dehydration in transgenic Arabidopsis. Plant Cell Rep.

[69] Lu M, Zhang D, Shi Y, Song Y, Wang T, Li Y (2013). Expression of SbSNAC1, a NAC transcription factor from sorghum, confers drought tolerance to transgenic Arabidopsis. Plant Cell Tiss Organ Cult.

[70] Wang Z, Rashotte AM, Moss AG, Dane F (2014). Two NAC transcription factors from Citrullus colocynthis, CcNAC1, CcNAC2 implicated in multiple stress responses. Acta Physiol Plant.

[71] Wang YX (2013). Characterization of a novel Medicago sativa NAC transcription factor gene involved in response to drought stress. Mol Biol Rep.

[72] Xu Q, He Q, Li S, Tian Z (2014). Molecular characterization of StNAC2 in potato and its overexpression confers drought and salt tolerance. Acta Physiol Plant.

[73] Mao H, Wang H, Liu S, Li Z, Yang X, Yan J, Li J, Tran LS, Qin F (2015). A transposable element in a NAC gene is associated with drought tolerance in maize seedlings. Nat Commun.

[74] Fujita Y, Fujita M, Shinozaki K, Yamaguchi-Shinozaki K (2011). ABA-mediated transcriptional regulation inresponse to osmotic stress in plants. J Plant Res.

[75] Finn RD, Coggill P, Eberhardt RY, Eddy SR, Mistry J, Mitchell AL, Potter SC, Punta M, Qureshi M, Sangrador-Vegas A, Salazar GA, Tate J, Bateman A (2016). The Pfam protein families database: towards a more sustainable future. Nucleic Acids Res.

[76] Finn RD, Clements J, Arndt W, Miller BL, Wheeler TJ, Schreiber F, Bateman A, Eddy SR (2015). HMMER web server: 2015 update. Nucleic Acids Res.

[77] Guo AY, Zhu QH, Chen X, Luo JC (2007). GSDS: a gene structure display server. Yi Chuan.

[78] Rombauts S, Déhais P, Van Montagu M, Rouzé P (1999). PlantCARE, a plant cis-acting regulatory element database. Nucleic Acids Res.

