# Supplementary figures and images for: Comprehensive analysis and discovery of drought-related NAC transcription factors in common bean
Source: BMC Plant Biol. 2016 Sep 7;16(1):193. doi: 10.1186/s12870-016-0882-5 (PMC5013670; doi:10.1186/s12870-016-0882-5)

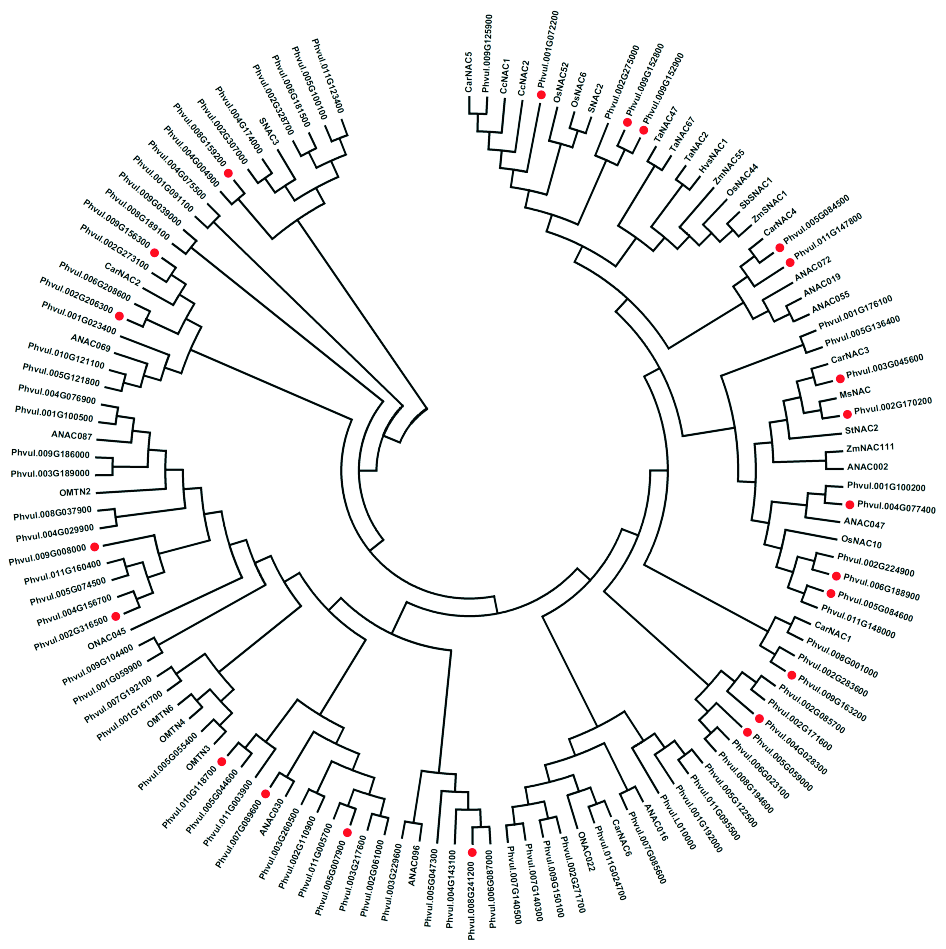

Supplement: Additional file 7: Figure S1. — Phylogenetic relationship of common bean NAC genes with drought-related NAC genes from other species. The phylogenetic tree was constructed in MEGA6.0 using the neighbor-joining method with 1000 bootstrap replicates. Each common bean drought-related NAC protein is indicated by a red dot. Sequences of drought-related NAC genes from other species downloaded from NCBI: CarNAC1(ACA96935), CarNAC2(ACA96936), CarNAC3(FJ356671), CarNAC4(ACS94037), CarNAC5(ACS94038), CarNAC6(ACS94039), CcNAC1(AHJ38168), CcNAC2(AHJ38169), SNAC2(XP_015620920), SNAC3(XP_015615070), ONAC022(AK107090), ONAC045(AK067922), OsNAC6(EAY76735), OsNAC10(XP_015645677), OsNAC44(AIQ84824), OsNAC52(AAT44250), OMTN2(XP_015633922), OMTN3(XP_015620576), OMTN4(XP_015643121), OMTN6(XP_015648318), TaNAC2(AAU08786), TaNAC47(KT345698), TaNAC67(KF646593), HvsNAC1(JF796130), ZmNAC55(AFW67212), ZmNAC111(NP_001183815), ZmSNAC1(AEY78612), SbSNAC1(AGG40203), MsNAC(HM237304), StNAC2(NP_001275015), ANAC002(At1g01720), ANAC016(At1g34180), ANAC019(At1g52890), ANAC030(At1g71930), ANAC047(At3g04070), ANAC055(At3g15500), ANAC069(At4g01550), ANAC072(At4g27410), ANAC087(At5g18270), ANAC096(At5g46590). (TIF 3509 kb) [file 12870_2016_882_MOESM7_ESM.tif]
